# Purification and partial genome characterization of the bacterial endosymbiont *Blattabacterium cuenoti *from the fat bodies of cockroaches

**DOI:** 10.1186/1756-0500-1-118

**Published:** 2008-11-25

**Authors:** Gaku Tokuda, Nathan Lo, Aya Takase, Akinori Yamada, Yoshinobu Hayashi, Hirofumi Watanabe

**Affiliations:** 1 Center of Molecular Biosciences (COMB), University of the Ryukyus, Nishihara, Okinawa 903-0213, Japan; 2 National Institute of Agrobiological Science, Tsukuba, Ibaraki 305-8634, Japan; 3 Australian Museum, Sydney, NSW 2010, Australia; 4 Laboratory of Forest Ecology, Graduate School of Agriculture, Kyoto University, Kyoto 606-8502, Japan

## Abstract

**Background:**

Symbiotic relationships between intracellular bacteria and eukaryotes are widespread in nature. Genome sequencing of the bacterial partner has provided a number of key insights into the basis of these symbioses. A challenging aspect of sequencing symbiont genomes is separating the bacteria from the host tissues. In the present study, we describe a simple method of endosymbiont purification from complex environment, using *Blattabacterium cuenoti *inhabiting in cockroaches as a model system.

**Findings:**

*B. cuenoti *cells were successfully purified from the fat bodies of the cockroach *Panesthia angustipennis *by a combination of slow- and fast-speed centrifugal fractionations, nylon-membrane filtration, and centrifugation with Percoll solutions. We performed pulse-field electrophoresis, diagnostic PCR and random sequencing of the shoutgun library. These experiments confirmed minimal contamination of host and mitochondrial DNA. The genome size and the G+C content of *B. cuenoti *were inferred to be 650 kb and 32.1 ± 7.6%, respectively.

**Conclusion:**

The present study showed successful purification and characterization of the genome of *B. cuenoti*. Our methodology should be applicable for future symbiont genome sequencing projects. An advantage of the present purification method is that each step is easily performed with ordinary microtubes and a microcentrifuge, and without DNase treatment.

## Background

Many eukaryotes have developed symbiotic relationships with bacterial endocytosymbionts [1,2]. These symbioses typically involve provision of nutrients by the bacterial partner to its host, and/or manipulation by the bacterial partner of the hosts' reproductive system [3]. Prior to the recent genomic revolution, details of their metabolism and physiology of many of these bacteria remained unknown, in part due to the difficulty of culturing these bacteria. Genome analyses have provided a wealth of information that will be crucial for elucidating the biology of these endosymbionts. The focus has been on symbionts in the phylum proteobacteria [4-13], although a few representatives of the other phyla have recently been sequenced [14,15].

A challenging step in sequencing symbiont genomes is purification of the bacterial cells. In some cases, bacterial DNA that was directly extracted from accumulated bacteriocytes was pure enough for sequencing analyses [5,9]. However, in other cases, it was necessary to remove contaminated DNA derived from host nuclei and/or mitochondria. For example, purification of *Blochmannia *and *Wolbachia *required the precise adjustment of treatment conditions of DNase I in order to remove DNA of contaminated organelles [6,16]; failure of this treatment caused complete digestion of the bacterial genome [16]. This problem was avoided during purification of *Buchnera *from aphids by use of Percoll-gradient centrifugation to separate the bacterial cells from other organelles or cellular debris [17]. An elegant method was applied to purify TG-1 bacteria from termite flagellates, where a single protozoan cell was manipulated and the posterior part of the protist cell was manually ruptured to collect the specific bacteria [15]. Additional examples of successful endosymbiont isolation from complex environments may facilitate the study of other bacterial symbioses.

Almost all cockroaches harbour the bacterial endocytosymbiont *Blattabacterium cuenoti *(phylum Bacteriodetes, class Flavobacteria) in mycetocytes of their fat bodies. Molecular phylogenetic studies show this bacterium is closely related to other Flavobacterial endosymbionts of insects, in particular *Sulcia muelleri *from auchenorrhynchan insects and male-kiling symbionts from ladybird beetles [18]. The *Blattabacterium*/cockroach symbiosis was first discovered in the late 19^th ^century [19]. These bacteria have been co-evolving with cockroaches for at least 130 million years [20,21]. It has been proposed that the symbiosis is mediated by juvenile hormone secreted from corpora allata [22]. On the basis of extensive biochemical, radiochemical, and antibiotic studies using normal and aposymbiotic cockroaches, the relationship has been shown to be one of obligate mutualism. The bacteria contribute to the nitrogen metabolism of their hosts by mobilizing the uric acid stored in the fat bodies when cockroaches feed on nitrogen-poor diets [23,24]. The uricolysis is thought to be mediated by xanthine dehydrogenase rather than uricase [23], although further details for this process have yet to be provided. In addition, the bacteria have been proposed to provide some essential amino acids to cockroaches [25] and to be involved in sulphate assimilation into sulphur amino acids [26]. These studies were conducted based on comparisons between normal and aposymbiotic insects, however, effects of the antimicrobial treatments on the intestinal bacterial diversity were not well assessed. Overall, the details of the interaction between the two partners have not yet been clarified.

Genomic or proteomic analyses are likely to greatly enhance our understanding of *B. cuenoti *biology. Purification of *B. cuenoti *cells is the first step in this process. Purification of *B. cuenoti *is challenging for the following reasons. First, the bacterial endosymbionts co-exist with organelles and nuclei that have to be eliminated. Second, in the fat body tissues, the mycetocyte is always surrounded by urocytes and trophocytes [27], which contain a large amount of urates, lipids, and other intracellular extra substances as well as usual organelles and nuclei. In the present paper, we describe the first successful purification of the genomic DNA of the flavobacterial endosymbiont in cockroaches.

## Methods

### Host insects

*Panesthia angustipennis *were collected at Mt. Tsukuba in Ibaraki prefecture, Japan. The cockroaches were reared with wood chips and sliced pieces of fresh carrot at room temperature.

### Purification of ***B. cuenoti*** from cockroaches

All procedures were performed at 4°C unless otherwise indicated. Two adult female individuals (6.5 g in total) were dissected to remove the fat bodies. The collected fat bodies were homogenized in 6 ml of a solution with the following components: 41.2 mM sodium chloride, 10.2 mM sodium hydrogen carbonate, 5.7 mM trisodium citrate and 14.5 mM potassium dihydrogenphosphate, and fixed in 2.5% formaldehyde for 2 h. The sample was centrifuged at 1700 × *g *for 40 min and the pellet was suspended in 2 ml of the same solution. The suspension was centrifuged at 300 × *g *for 2 min to remove the precipitates. The supernatant was centrifuged at 5000 × *g *for 20 min and the pellet was suspended in 1.5 ml of the same solution. This centrifugal step was repeated three times. The final suspension was filtered through nylon membranes of pore sizes 90 μm and 25 μm to remove the remaining large debris. The filtrate was centrifuged at 5000 × *g *for 20 min and the pellet was suspended in 500 μl of the initial solution. To separate the bacterial cells from mitochondria and the host nuclei, the suspension was overlaid on 5 ml of 30% Percoll solution (containing 5.5% PEG6000, 1.1% Ficoll, and 278 mM sucrose), which had been overlaid on 5 ml of 70% Percoll solution (containing 5% PEG6000, 1% Ficoll, and 250 mM sucrose) in advance. The sample was centrifuged at 12,000 × *g *for 20 min, and bacterial cells present between the 30% and 70% Percoll phases were collected. Alternatively, 50 μl of the suspension was overlaid on 500 μl of the 30% Percoll solution and centrifuged at 12,000 × *g *for 20 min. Pellet was suspended in 50 μl of 0.2 μm filter-sterilized distilled water and overlaid on 500 μl of the 70% Percoll solution. After centrifugation at 12,000 × *g *for 20 min, bacterial cells remained on the 70% Percoll solution were collected. The collected bacterial cells were washed with five volumes of the initial solution or filter-sterilized distilled water and centrifuged to form a bacterial pellet. An aliquot of the bacterial cells was stained with DAPI (4',6'-diamidino-2-phenylindole) and observed with an epifluorescence microscope.

### Pulse-field gel electrophoresis (PFGE)

Agarose embedded DNA (plug) was prepared using a CHEF bacterial genomic DNA plug kit (Bio-Rad, Hercules, CA, USA). The plug was extensively washed with TE and treated with 200 U of the homing enzyme I-*Ceu *I (New England Biolabs, Bevely, MA, USA) in 500 μl of 1 × digestion buffer (50 mM potassium acetate, 20 mM Tris-acetate, 10 mM magnesium acetate, 1 mM dithiothreitol (DTT), and 100 μg/ml bovine serum albumin, pH 7.9) to linearize the genomic DNA by cutting the prokaryotic 23S rRNA gene. The plug was briefly rinsed with 0.5% SDS in 0.5 × TBE (1 × TBE consists of 0.89 M Tris-HCl, 0.89 M boric acid, and 20 mM EDTA, pH 8.3) and applied to PFGE.

The plug was embedded in a contour-clamped homogeneous electric field (CHEF) gel (1%) and the bacterial DNA was run using a CHEF-DRII system (Bio-Rad) at 10–15°C. The electrophoresis was carried out with a pulse switching time of every 60 s over 15 h, then every 90 s over 9 h in 0.5 × TBE (as running buffer) at 200 V (6 V/cm). To check the presence or absence of DNA fragments less than 250 kb in length, electrophoresis was performed with gradual increase of the pulse switching time from 30 s to 90 s over 24 h at 150 V. The presence or absence of DNA fragments less than 10 kb was checked by conventional electrophoresis with a 1% agarose gel. The gels were stained with ethidium bromide and observed on a UV trans-illuminator.

To confirm the genome size, the plug was treated with 200 U of the restriction enzyme *Ksp *I (= *Sac *II or *Sst *II) (Roche, Penzberg, Germany) in 500 μl of 1 × digestion buffer (10 mM Tris-HCl, 10 mM magnesium chloride, and 1 mM dithioerythritol, pH 7.5). Pulse-Field electrophoresis was performed at 6 V/cm using ramped pulse times from 5 to 20 s for 18 h, followed by a pulse switching time of every 60 s over 15 h, then every 90 s over 6 h in 0.5 × TBE.

### DNA purification and diagnostic PCR

To check if the DNA band actually originated from the purified *B. cuenoti*, the band was excised from the gel and washed with 1 × β-Agarase I Reaction Buffer (10 mM Bis Tris-HCl, 1 mM EDTA, pH 6.5) (New England Biolabs). The agarose block containing the DNA band was completely melted at 90°C and was cooled to 50°C. Then, β-Agarase I (New England Biolabs) was added to be 20 U/ml and the sample was incubated at 45°C for 1 h. The bacterial genomic DNA was extracted using a conventional phenol/chloroform method [28]. The genomic DNA collected with ethanol precipitation was dissolved in 50 μl of sterilized distilled water, which was used as PCR template.

Diagnostic PCR was performed using forward (5'-GAT GGC GAC CGG CGT ACG GGT GCG-3', positions corresponding to 45-68 of Genbank AB231604) and reverse (5'-TAC ACC ACA CAT TCC AGC TAC TCC-3', positions corresponding to 641-618 of AB231604) primers specific for 16S rDNA of *B. cuenoti*, A-tLEU and B-tLYS [29] specific for mitochondrial cytochrome oxidase II, and newly designed forward (5'-AAA TTA CCC ACT CCC GGC AC-3', positions corresponding to 3-22 of Genbank AB036190) and reverse (5'-TGG TGC CCT TCC GTC AAT TC-3', positions corresponding to 829-810 of AB036190) primers specific for 18S rDNA of *P. angustipennis*, respectively. The temperature regimen for 30 cycles was 94°C for 30 s, 52°C for 30 s, and 72°C for 1 min. The amplified fragment size was confirmed by 1% agarose gel electrophoresis.

### Amplification of the whole genome and construction of shotgun library

To obtain the sufficient amount of DNA, the total genomic DNA extracted from the CHEF gel was amplified with a proofreading phi29 DNA polymerase using a GenomiPhi DNA amplification kit (GE Healthcare, Buckinghamshire, UK). The shotgun library (~2 kb) was constructed with a modification of the previous method [5]. Briefly, the amplified DNA was hydrodynamically cut (~2 kb) with the hydroshear DNA shearer (Digilab Genomic Solutions, Ann Arbor, MI). The fragmented DNA was treated with DNA polymerase I and blunt-ended with T4 DNA polymerase. After conventional agarose gel electrophoresis, DNA fragments were excised from the gel at the area of 2 kb in size and purified. The obtained DNA fragments were ligated at the *Hinc *II site of phospholylated pUC118 plasmid vector and transformed into *Escherichia coli *DH10B. 96 clones were randomly selected and one-pass sequences were determined using an ABI 3700 sequencer.

## Results and discussion

### Development of the purification method of *B. cuenoti *cells

**Figure 1:**
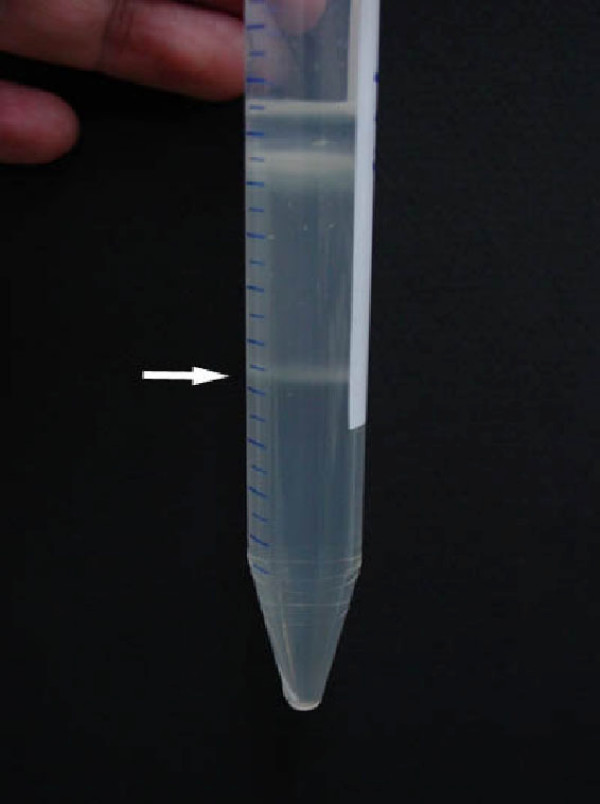
**Purification of *B. cuenoi *cells by two-layered Percoll centrifugation**. *B. cuenoti *cells (arrow) between two layers of the Percoll solutions after centrifugation at 12,000 × *g *for 20 min.

**Figure 2:**
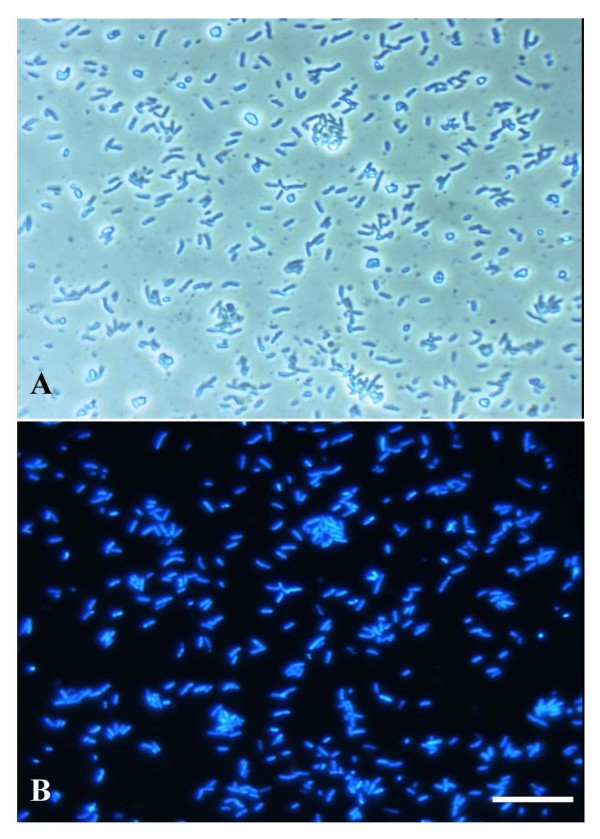
**Purified bacterial cells of *B. cuenoti***. Light micrograph of purified *B. cuenoti*. B. Fluorescent image of DAPI-stained *B. cuenoti*, which is the same field as that shown as (a). Bar, 25 μm.

A challenge in genomic analyses of bacterial endosymbionts is purification of bacterial cells. Thus, we aimed to develop a simple purification method for endocytosymbionts from a complex environment. Unlike the case for some insects, it is not possible to manually isolate bacteriocytes from the fat bodies of cockroaches. Thus, we employed four purification steps to obtain the pure *B. cuenoti*. The first step was to collect only fat bodies from the cockroaches by dissection. The structure of *B. cuenoti *cells throughout the purification steps was aided by immediate fixation of the fat body homogenate with paraformaldehyde. The second step was a combination of fast- and slow-speed centrifugal fractionations to separate *B. cuenoti *from larger and smaller cellular or cytosolic debris and lipids. The third step was filtration of the centrifuged homogenate with nylon membranes to eliminate remaining larger debris from the sample. However, these steps did not separate the bacterial cells from mitochondria and host nuclei. Therefore, to purify the bacterial cells without DNase treatment, we initially tried Percoll gradient centrifugation based on the purification method of *Buchnera *[17] as well as an autogradient formation with Percoll using an ultracentrifuge. However, the bacterial band was not formed in the gradient solutions. Instead of the gradient centrifugation, we employed a two-layered Percoll centrifugation, where the concentrations of the ingredients in the Percoll solutions were precisely adjusted during preliminary experiments. This method eventually resulted in a white band between the upper (30%) and bottom (70%) Percoll layers (Fig. 1). Light microscopic observation revealed that the purified sample consisted of short bacterial rods with a slight contamination of small particles (Fig. 2A). However, DAPI signals were only detected from the short rods, thus contaminations of neither mitochondria nor nuclei were observed (Fig. 2B). The short rods ranged in length from approximately 2 to 5 μm and often formed binary fission pairs (Fig. 2B). These morphologies are consistent with those of the previously reported *Blattabactterium *[30,31], suggesting the successful purification of the endosymbiont. We also found that the two-layered Percoll centrifugation was not mandatory. Centrifugation with 30% Percoll solution in a microtube followed by centrifugation with the 70% solution produced the same result as Fig. 2. Stepwise changes in the concentration of the Percoll solutions with microtubes may enable to this method to be applied to other endosymbionts living in similarly complex host environments.

### Genome size estimation and purity confirmation

**Figure 3:**
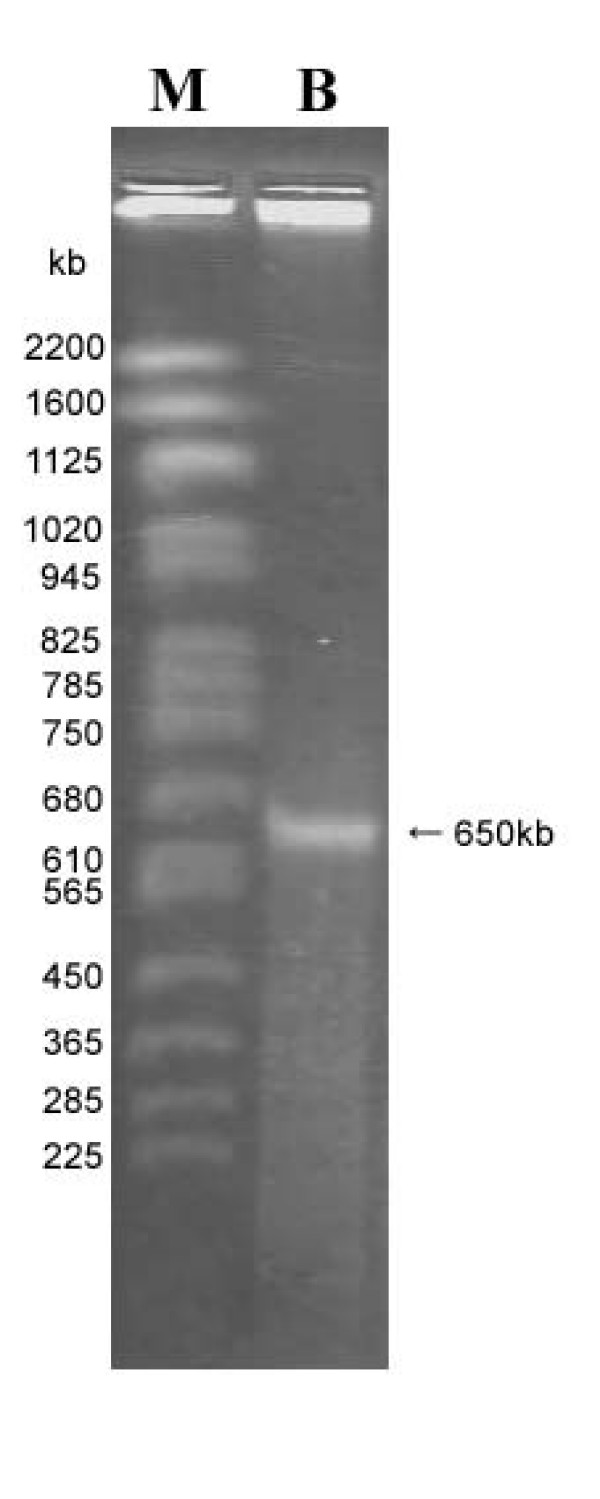
**Electrophoretogram of the genomic DNA from *B. cuenoti *in the CHEF gel**. M: Marker DNA of *S. cerevisiae *chromosomes (Bio-Rad). B: Genomic DNA of *B. cuenoti*. Arrow indicates the DNA band observed.

To achieve complete purification of the bacterial DNA, we performed pulse-field gel electrophoresis (PFGE) using plugs prepared from the purified bacterial cells. If the genome is circular, the DNA does not migrate smoothly in CHEF gels during PFGE [16]. In the present study, linearization of the bacterial chromosome with an enzymatic cleavage was necessary prior to electrophoresis. Although we initially tried to digest the DNA with the restriction enzyme *Not *I (that recognizes an 8-bp motif rich in G and C), no bands were observed. Thus, we treated the bacterial DNA with the homing endonuclease I-*Ceu *I, that cleaves many prokaryotic 23S rRNA genes at only one specific site [32]. Fig. 3 shows the electrophoretogram of the genomic DNA from *B. cuenoti*. The electrophoresis resulted in only one band; the absence of other bands was confirmed under other PFGE conditions and conventional submarine electrophoresis. The results suggest that *B. cuenoti *possesses only one copy of the ribosomal RNA gene complement (i.e. 5S, 16S, and 23S rDNAs) on a circular genome, as is the case of the majority of other bacterial endosymbionts (i.e. *Buchnera *spp., *Blochmannia *spp., and *Carsonella ruddii*). The size of the bacterial genomic DNA was estimated to be 650 kb by comparing its mobility with chromosomal fragments of *Saccharomyces cerevisiae *on PFGE (Fig. 3). This result was further confirmed with the other restriction enzymes, *Ksp *I, which produced two fragments (see Additional file 1). No evidence for extrachromosomal plasmids was found.

**Figure 4:**
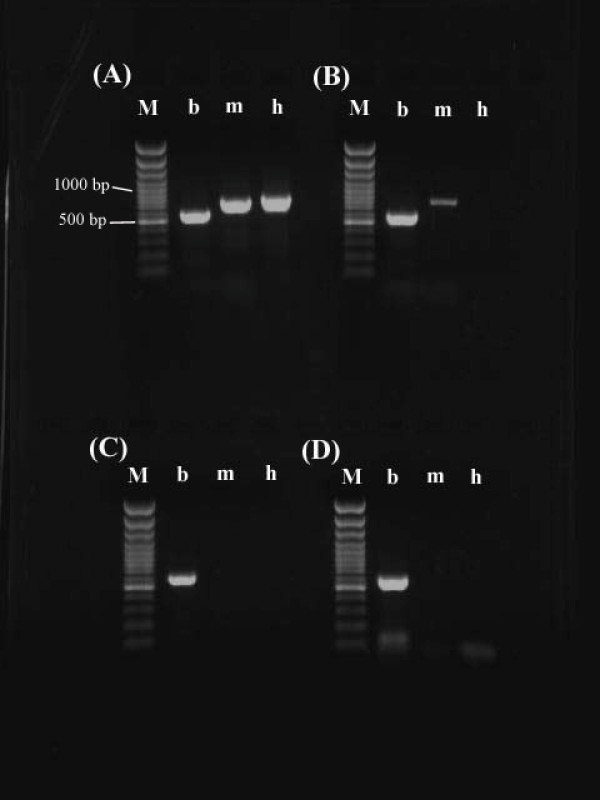
**Diagnostic PCR using DNA extracted during purification of the bacterial endosymbiont**. A. When PCR was performed using a template prepared from the fat bodies, DNA fragments of *B. cuenoti *(b), mitochondria (m) and host *Panesthia *nuclei (h) were observed. B. Although the host DNA was disappeared after the Percoll centrifugation, only a trace of contamination of mitochondrial DNA was amplified. C. When the *B. cuenoti *DNA was digested with I-*Ceu *I separated using PFGE, no contaminations of mitochondrial and host's DNAs were detected. D. As PCR cycles were increased to be 35 cycles, no contaminations were detectable. M: 100-bp ladder.

To check the origin of the DNA band, PCR was performed using primers specific for the *B. cuenoti *16S rDNA, cockroach 18S rDNA, and mitochondrial COII. Contamination of mitochondrial or host nuclear DNA was significantly removed from the sample collected after the Percoll centrifugation and eventually not detected after PFGE (Fig. 4). Since only a trace of amplification of contaminated DNA was detected without the Percoll centrifugation (Fig. 4B), PFGE separation of genomic DNA may be only necessary when very pure DNA is required for further experiments.

So far, intracellular endosymbiont genome sequencing projects have primarily focused on members of the proteobacteria phylum (Table 1). The genomes of these endosymbionts range from 450 kb to 1.7 Mb (Table 1), with the exception of the psyllids bacterial symbiont (160 kb) [9], the secondary flavobacterial endosymbiont of sharpshooters (245 kb) [14], and the secondary endosymbiont of tsetse flies (4.2 Mb) [33]. From comparisons with their free-living relatives, it is widely accepted that the intracellular symbionts have lost significant amounts of genomic information since adopting the intracellular lifestyle [3]. Indeed, the secondary endosymbiont of tsetse flies, *Sodalis glossinidius*, which was recently diverged from a free-living ancestor, shows a large genome size (4.2 Mb) and massive slow erosion at individual loci [33]. Free-living and parasitic relatives in Flavobacteria have genome sizes ranging from 2.7 to 6 Mb (Table 1), compared with 650 kb for *B. cuenoti*. Thus *B. cuenoti *is likely to have lost a significant number of genes since its ancestors first entered into a symbiotic relationship with insects. The present study is the second demonstration of a Bacteroidetes symbiont with a reduced genome, the first being *Sulcia muelleri *(245 kb) [14]. While *B. cuenoti *is a primary symbiont, *S. muelleri *co-inhabits sharpshooter cells with the primary endosymbiont *Baumannia cicadellinicola *[14]. Very recently, the small genome (1.1 Mb) of a bacterial endosymbiont (phylum TG-1) of termite flagellates has been determined [15]. In addition, similar genome reduction has also been reported from commensal and parasitic microbes such as those belonging to chlamydiae, rickettsiae, and mollicutes [34,35]. These findings strongly suggest evolutionary plasticity of bacterial genomes in response to their lifestyles and endosymbiotic genome reduction as a phenomenon that occurs across different bacterial phyla.

**Table 1:** Comparison of genome sizes among free-living, pathogenic and endosymbiotic bacteria

**Bacterial species (host or origin)**	**Genome size (Reference)**
**Bacteriodetes, class Flavobacteria**	
*Flavobacterium johnsoniae *(free-living)	~6 Mb (Genbank AAPM00000000)
*Leeuwenhoekiella blandensis *(free-living)	4.24 Mb [36]
*Polaribacter irgensii *(free-living)	2.74 Mb [36]
*F. Psychrophilum *(a fish pathogen)	2.86 Mb [37]
*Sulcia muelleri *(sharpshooters)	245 kb [14]
*Blattabacterium cuenoti *(cockroaches)	650 kb **(this study)**
	
**Proteobacteria Endosymbionts**	
*Baumannia cicadellinicola *(sharpshooters)	686 kb [11]
*Blochmannia floridanus *(carpenter ants)	705 kb [6]
*Buchnera *spp. (pea arphids)	416–640 kb [4,10]
*Carsonella ruddii *(psyllids)	160 kb [9]
*Sodalis glossinidius *(tsetse flies)	4.17 Mb [33]
*Wigglesworthia glossinidia *(tsetse flies)	698 kb [5]
*Wolbachia *spp. (insects and nematodes)	0.95–1.66 Mb [7,8,16]
*Caryptogena *symbiont (deep-sea clams)	1.02 Mb [12]
*Riftia magnifica *(deep-sea clams)	1.16 Mb [13]
	
**Termite Group I Endosymbiont**	
*Trichonympha *symbiont (termite flagellates)	1.13 Mb [15]

In order to confirm that the genomic DNA was pure enough for further applications, we constructed the shotgun library and randomly selected 96 clones were one-pass sequenced (see Additional file 2). Except for 6 clones that possessed vector-derived contaminants, only one clone showed apparent contamination of the host genomic DNA, while no mitochondrial DNA was detected. The average G+C content of the DNA fragments (excluding the host and contaminated vector DNA fragments) was 32.1 ± 7.6% (see Additional file 2), which is consistent with many other reduced genomes of endosymbionts showing relatively low G+C content ranging from 16.6 to 35% [12,14]. The genomes of free-living Bacteroidetes species typically have more than 40% G+C content [14].

The present method will facilitate genome sequence analysis of *B. cuenoti*, thereby providing important information on which genes have been retained by the symbiont to contribute to the host's metabolism. The ability to break down uric acid, one of the most intriguing functions predicted in *B. cuenoti*, is rare among members of *Flavobacterium *genus, which represent the closest free-living relatives. It will thus be interesting to see the phylogenetic affinity of uricolytic genes in *B. cuenoti*, if they are present in the genome.

## Competing interests

The authors declare that they have no competing interests.

## Authors' contributions

GT, NL, AY, and HW designed the research project. GT, AT, and HW collected the insects. GT, AT and YH performed the purification and PFGE experiments. GT annotated the sequences of the shotgun library. GT and NL wrote the paper. All authors approved and read the final manuscript.

## Supplementary Material

Additional file 1 **Fig. S1 Electrophoretogram of the genomic DNA from *B. cuenoti *in the CHEF gel**. The genomic DNA was cut with *Ksp *I. The sum of the restricted fragment sizes was consistent with the result shown as Fig. 3. M1: Marker DNA of *S. cerevisiae *chromosomes. M2: MidRange PFG Marker I (New England Biolabs). B: Genomic DNA of *B. cuenoti*. Click here for file

Additional file 2 **Table S1 Sequencing analysis of the shotgun library clones**. Click here for file

## References

[B1] Buchner P (1965). Endosymbiosis of animals with plant microorganisms.

[B2] Wernegreen JJ (2002). Genome evolution in bacterial endosymbionts of insects. Nat Rev Genet.

[B3] Dale C, Moran NA (2006). Molecular interactions between bacterial symbionts and their hosts. Cell.

[B4] Shigenobu S, Watanabe H, Hattori M, Sakaki Y, Ishikawa H (2000). Genome sequence of the endocellular bacterial symbiont of aphids *Buchnera *sp. APS. Nature.

[B5] Akman L, Yamashita A, Watanabe H, Oshima K, Shiba T, Hattori M, Aksoy S (2002). Genome sequence of the endocellular obligate symbiont of tsetse flies, *Wigglesworthia glossinidia*. Nat Genet.

[B6] Gil R, Silva FJ, Zients E, Delmotte F, González-Candelas F, Latorre A, Rausell C, Kamerbeek J, Gadau J, Hölldobler B, van Ham RCHJ, Gross R, Moya A (2003). The genome sequence of *Blochmannia floridanus*: comparative analysis of reduced genomes. Proc Natl Acad Sci USA.

[B7] Wu M, Sun LV, Vamathevan J, Riegler M, Deboy R, Brownlie JC, McGraw EA, Martin W, Esser C, Ahmadinejad N, Wiegand C, Madupu R, Beanan MJ, Brinkac LM, Daugherty SC, Durkin AS, Kolonay JF, Nelson WC, Mohamoud Y, Lee P, Berry K, Young MB, Utterback T, Weidman J, Nierman WC, Paulsen IT, Nelson KE, Tettelin H, O'Neill SL, Eisen JA (2004). Phylogenomics of the reproductive parasite *Wolbachia pipientis *wMel: a streamlined genome overrun by mobile genetic elements. PLoS Biol.

[B8] Foster J, Ganatra M, Kamal I, Ware J, Makarova K, Ivanova N, Bhattacharyya A, Kapatral V, Kumar S, Posfai J, Vincze T, Ingram J, Moran L, Lapidus A, Omelchenko M, Kyrpides N, Ghedin E, Wang S, Goltsman E, Joukov V, Ostrovskaya O, Tsukerman K, Mazur M, Comb D, Koonin E, Slatko B (2005). The *Wolbachia *Genome of *Brugia malayi*: endosymbiont evolution within a human pathogenic nematode. PLoS Biol.

[B9] Nakabachi A, Yamashita A, Yoh H, Ishikawa H, Dunbar HE, Moran NA, Hattori M (2006). The 160-kilobase genome of the bacterial endosymbiont *Carsonella*. Science.

[B10] Pérez-Brocal V, Gil R, Ramos R, Lamelas A, Postigo M, Michelena JM, Silva FJ, Moya A, Latorre A (2006). A small microbial genome: the end of a long symbiotic relationship?. Science.

[B11] Wu D, Daugherty SC, Van Aken SE, Pai GH, Watkins KL, Khouri H, Tallon LJ, Zaborsky JM, Dunbar HE, Tran PL, Moran NA, Eisen JA (2006). Metabolic complementarity and genomics of the dual bacterial symbiosis of sharpshooters. PLoS Biol.

[B12] Kuwahara H, Yoshida T, Takaki Y, Shimamura S, Nishi S, Harada M, Matsuyama K, Takishita K, Kawato M, Uematsu K, Fujiwara Y, Sato T, Kato C, Kitagawa M, Kato I, Maruyama T (2007). Reduced genome of the thioautotrophic intracellular symbiont in a deep-sea clam, *Calyptogena okutanii*. Curr Biol.

[B13] Newton ILG, Woyke T, Auchtung TA, Dilly GF, Dutton RJ, Fisher MC, Fontanez KM, Lau E, Stewart FJ, Richardson PM, Barry KW, Saunders E, Detter JC, Wu D, Eisen JA, Cavanaugh CM (2007). The *Calyptogena magnifica *chemoautotrophic symbiont genome. Science.

[B14] McCutcheon JP, Moran NA (2007). Parallel genomic evolution and metabolic interdependence in an ancient symbiosis. Proc Natl Acad Sci USA.

[B15] Hongoh Y, Sharma VK, Prakash T, Noda S, Taylor TD, Kudo T, Sakaki Y, Toyoda A, Hattori M, Ohkuma M (2008). Complete genome of the uncultured Termite Group 1 bacteria in a single host protist cell. Proc Natl Acad Sci USA.

[B16] Sun LV, Foster JM, Tzertzinis G, Ono M, Bandi C, Slatko BE, O'Neill S (2001). Determination of *Wolbachia *genome size by pulse-field gel electrophoresis. J Bacteriol.

[B17] Charles H, Ishikawa H (1999). Physical and genetic map of the genome of *Buchnera*, the primary endosymbiont of the pea aphid Acyrthosiphon pisum. J Mol Evol.

[B18] Gruwell ME, Morse GE, Normark BB (2007). Phylogenetic congruence of armored scale insects (Hemiptera: Diaspididae) and their primary endosymbionts from the phylum Bacteroidetes. Mol Phylogenet Evol.

[B19] Blochmann F (1887). Vorkommen bacterienähnliche Köpperchen in den Geweben und Eiern verschiedener Insekten. Biologisches Centralblatt.

[B20] Bandi C, Sironi M, Damiani G, Magrassi L, Nalepa CA, Laudani U, Sacchi L (1995). The establishment of intracellular symbiosis in an ancestor of cockroaches and termites. Proc R Soc Lond B Biol Sci.

[B21] Lo N, Bandi C, Watanabe H, Nalepa C, Beninati T (2003). Evidence for cocladogenesis between diverse dictyopteran lineages and their intracellular endosymbionts. Mol Biol Evol.

[B22] Philippe C, Latge JP, Prevost MC (1988). *In vitro *characterization of hormonal action on *Periplaneta americana *endocytobiotes. Endocytobiosis and Cell Research.

[B23] Cochran DG (1985). Nitrogen excretion in cockroaches. Ann Rev Entomol.

[B24] Wren HN, Cochran DG (1987). Xanthine dehydrogenase activity in the cockroach endosymbiont *Blattabacterium cuenoti *(Mercier 1906) Hollande and Favre 1931 and in the cockroach fat body. Comp Biochem Physiol.

[B25] Henry SM (1962). The significance of microorganisms in the nutrition of insects. Trans N Y Acad Sci.

[B26] Henry SM, Block RJ (1960). The sulphur metabolism of insects. IV. The conversion of inorganic sulphate to organic sulphur compounds in cockroaches. Contributions to Boyce Thompson Institute.

[B27] Bandi C, Sacchi L, Abe T, Bignell DE, Higashi M (2000). Intracellular symbiosis in termites. Termites: Evolution, Sociality, Symbioses, Ecology.

[B28] Sambrook J, Fritsch EF, Maniatis T (1989). Molecular cloning: a laboratory manual.

[B29] Maekawa K, Lo N, Kitade O, Miura T, Matsumoto T (1999). Molecular phylogeny and geographic distribution of wood-feeding cockroaches in East Asian islands. Mol Phylogenet Evol.

[B30] Brooks MA (1970). Comments on the classification of intracellular symbiotes of cockroaches and a description of the species. J Invertebr Pathol.

[B31] Dasch GA, Weiss E, Chang K, Krieg NR, Holt JG (1984). Endosymbionts in insects. Bergey's Manual of Systematic Bacteriology.

[B32] Liu SL, Hessel A, Sanderson KE (1993). Genomic mapping with I-*Ceu *I, an intron-encoded endonuclease specific for genes for ribosomal RNA, in *Salmonella *spp., *Escherichia coli*, and other bacteria. Proc Natl Acad Sci USA.

[B33] Toh H, Weiss BL, Perkin SAH, Yamashita A, Oshima K, Hattori M, Aksoy S (2006). Massive genome erosion and functional adaptations provide insights into the symbiotic lifestyle of *Sodalis glossinidius *in the tsetse host. Genome Res.

[B34] Palmer GH (2002). The highest priority: what microbial genomes are telling us about immunity. Vet Immunol Immunopathol.

[B35] Trachtenberg S (2005). Mollicutes. Curr Biol.

[B36] Pinhassi J, Bowman JP, Nedashkovskaya OI, Lekunberri I, Gomez-Consarnau L, Pedrós-Alió C (2006). *Leeuwenhoekiella blandensis *sp. nov., a genome-sequenced marine member of the family *Flavobacteriaceae*. Int J Syst Evol Microbiol.

[B37] Duchaud E, Boussaha M, Loux V, Bernardet J, Michel C, Kerouault B, Mondot S, Nicolas P, Bossy R, Caron C, Bessières P, Gibrat J-F, Claverol S, Dumetz F, Hénaff ML, Benmansour A (2007). Complete genome sequence of the fish pathogen *Flavobacterium psychrophilum*. Nat Biotechnol.

