# Supplementary figures and images for: Purification and partial genome characterization of the bacterial endosymbiont Blattabacterium cuenoti from the fat bodies of cockroaches
Source: BMC Res Notes. 2008 Nov 25;1:118. doi: 10.1186/1756-0500-1-118 (PMC2621225; doi:10.1186/1756-0500-1-118)

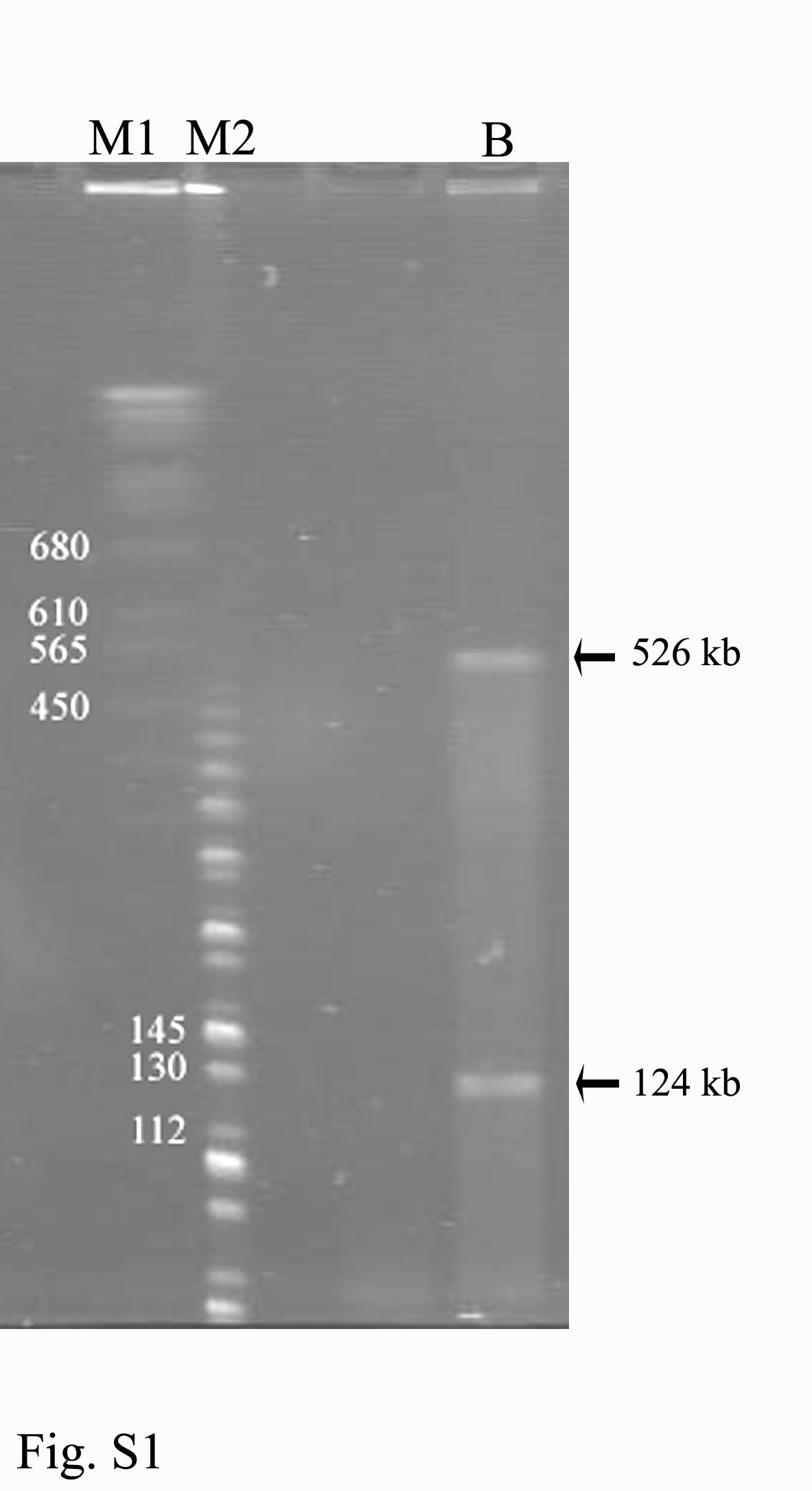

Supplement: Additional file 1 — Fig. S1 Electrophoretogram of the genomic DNA from B. cuenoti in the CHEF gel. The genomic DNA was cut with Ksp I. The sum of the restricted fragment sizes was consistent with the result shown as Fig. 3. M1: Marker DNA of S. cerevisiae chromosomes. M2: MidRange PFG Marker I (New England Biolabs). B: Genomic DNA of B. cuenoti. [file 1756-0500-1-118-S1.jpeg]
